# Supplementary figures and images for: Quantitative Analyses and Validation of Phospholipids and Sphingolipids in Ischemic Rat Brains
Source: Metabolites. 2022 Nov 6;12(11):1075. doi: 10.3390/metabo12111075 (PMC9694501; doi:10.3390/metabo12111075)

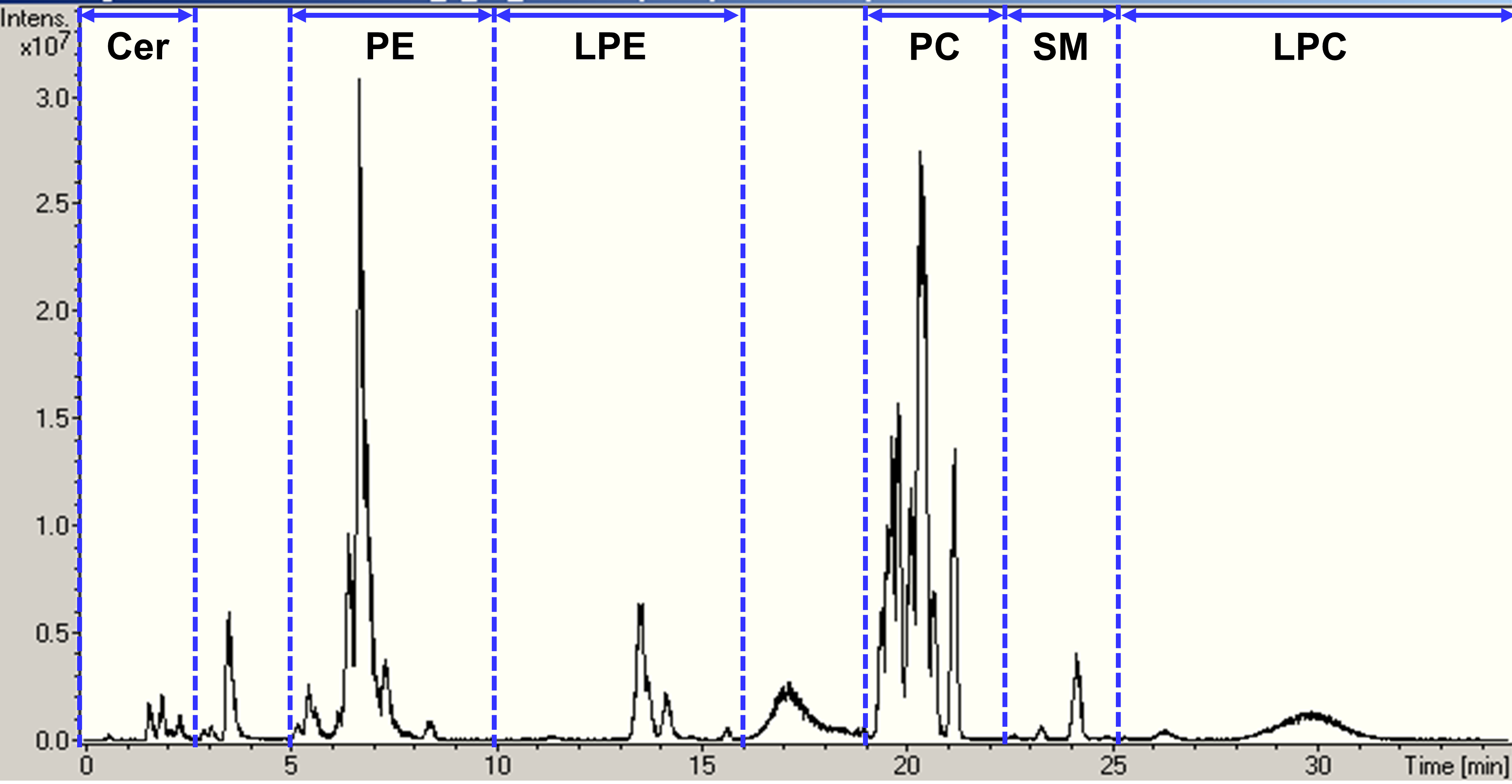

Supplement: Supplementary file 1 [file metabolites-12-01075-s001.zip › Supplementary_Figure_S1.tif]
